# Temporal changes in Weddell seal dive behavior over winter: Are females increasing foraging effort to support gestation?

**DOI:** 10.1002/ece3.4643

**Published:** 2018-11-23

**Authors:** Michelle R. Shero, Kimberly T. Goetz, Daniel P. Costa, Jennifer M. Burns

**Affiliations:** ^1^ Biology Department Woods Hole Oceanographic Institution Woods Hole Massachusetts; ^2^ Department of Biological Sciences University of Alaska Anchorage Anchorage Alaska; ^3^ National Institute of Water and Atmospheric Research Wellington New Zealand; ^4^ Department of Ecology and Evolutionary Biology University of California Santa Cruz Santa Cruz California

**Keywords:** aerobic capacity, aerobic dive limit, dive behavior, gestation, marine mammals, pinniped, pregnancy, reproduction

## Abstract

In capital‐breeding marine mammals, prey acquisition during the foraging trip coinciding with gestation must provide energy to meet the immediate needs of the growing fetus and also a store to meet the subsequent demands of lactation. Weddell seals (*Leptonychotes weddellii*) that give birth following the gestational (winter) foraging period gain similar proportions of mass and lipid as compared to females that fail to give birth. Therefore, any changes in foraging behavior can be attributed to gestational costs. To investigate differences in foraging effort associated with successful reproduction, twenty‐three satellite tags were deployed on post‐molt female Weddell seals in the Ross Sea. Of the 20 females that returned to the area the following year, 12 females gave birth and eight did not. Females that gave birth the following year began the winter foraging period with significantly longer and deeper dives, as compared to non‐reproductive seals. Mid‐ to late winter, reproductive females spent a significantly greater proportion of the day diving, and either depressed their diving metabolic rates (DMR), or exceeded their calculated aerobic dive limit (cADL) more frequently than females that returned without a pup. Moreover, non‐reproductive females organized their dives into 2–3 short bouts per day on average (BOUT_short_; 7.06 ± 1.29 hr; mean ± 95% CI), whereas reproductive females made 1–2 BOUT_short_ per day (10.9 ± 2.84 hr), comprising one long daily foraging bout without rest. The magnitude of the increase in dive activity budgets and depression in calculated DMR closely matched the estimated energetic requirements of supporting a fetus. This study is one of the first to identify increases in foraging effort that are associated with successful reproduction in a top predator and indicates that reproductive females must operate closer to their physiological limits to support gestational costs.

## 1 INTRODUCTION

The survival and reproductive output of wild animals depends on the individual's ability to acquire sufficient resources and energy in the face of changing ambient conditions and ephemeral prey, and such challenges are accentuated in high‐latitude, polar environments that experience dramatic seasonality (Bluhm & Gradinger, 2008; Bronson, 2009; Croxall, 1992; Walsh, 2008). In mammals, supporting reproduction comes with large costs, including increased foraging effort, enhanced vigilance and predator avoidance, shifts in habitat range, and reduced survivorship (Barclay, 1989; Berger, 1991; Ciuti, Bongi, Vassale, & Apollonio, 2006; Desprez, Gimenez, McMahon, Hindell, & Harcourt, 2018; Hadley, Rotella, & Garrott, 2007; Henry, Thomas, Vaudry, & Carrier, 2002). Reproductive costs are due to high energetic demands during both prenatal (gestational) and postnatal (lactation) maternal investment periods (Brody, 1945; Costa, 1991; Gittleman & Thompson, 1988). To support the additional demands of reproduction, animals must appropriately allocate acquired energy between pathways supporting immediate costs (i.e., female maintenance, fetal growth), and those promoting tissue accretion to store on‐board for later use (i.e., capital reserves). Larger energetic reserves can be deposited by increasing caloric intake and/or depressing metabolic rates (Speakman & Rowland, 1999). Among mammals, many marine species have evolved the ability to store such large energetic capital that many critical life history events can be spatially and temporally separated from prey resources, referred to as a capital‐breeding strategy (Boness & Bowen, 1996; Costa, 1991; Schulz & Bowen, 2004).

In association with parturition, nursing, and molting, many marine mammals experience drastic changes in activity budgets and dramatic weight fluctuations (Castellini & Rea, 1992; Castellini, Davis, & Kooyman, 1992; Costa, Boeuf, Ortiz, & Huntley, 1986; Crocker, Champagne, Fowler, & Houser, 2014; Crocker, Williams, Costa, & Boeuf, 2001; McDonald, Crocker, Burns, & Costa, 2008; Thompson, Fedak, McConnell, & Nicholas, 1989; Wheatley, Bradshaw, Davis, Harcourt, & Hindell, 2006). This is because the timing of reproduction is often constrained by environmental conditions and must be short in duration. After depleting energetic capital to support critical life history events, animals must recuperate mass and condition efficiently, for the next year's reproductive efforts (Carlini, Daneri, Marquez, Soave, & Poljak, 1997), and short‐ and long‐term climate regime shifts that impact local productivity exhibit strong linkages with population pupping rates (Chambert, Rotella, & Garrott, 2012; Hindell et al., 2017; Paterson, Rotella, Arrigo, & Garrott, 2015). Therefore, greater investment of resources or inadequate recovery of energy stores is likely to influence the balance between current and future reproductive success, in which the energy and time devoted to the current reproductive event can impact expected future fecundity due to carry‐over costs (Boggs, 1992; Desprez et al., 2018; McMahon, Harcourt, Burton, Daniel, & Hindell, 2017; Stearns, 1992). Particularly for capital‐breeding species, foraging success during the gestation period has the potential to impact whether pregnancies are carried to full‐term, how much capital is available to support lactation (Crocker et al., 2001; McMahon et al., 2017; Wheatley et al., 2006), and how likely the pup is to survive through the first year (Proffitt, Garrott, & Rotella, 2008; Proffitt, Garrott, Rotella, & Wheatley, 2007). However, relatively few studies have quantified the additional diving and foraging effort necessary to produce a pup.

Changes in foraging effort can be driven by variation in the animal's energetic demands, underlying physiology, or fluctuations in the abundance or predictability of prey. Any of these pathways can influence the types of foraging activities that produce the highest rates of energy gain (Houston & Carbone, 1992; Kramer, 1988; Thompson & Fedak, 2001). In addition, the diving capabilities of marine mammals are constrained by the magnitude and management (i.e., diving metabolic rate, DMR) of endogenous oxygen (O_2_) stores while animals are underwater (Butler & Jones, 1997; Hochachka & Storey, 1975; Kooyman & Ponganis, 1998), with the vast majority of dives remaining aerobic in nature (Kooyman, Wahrenbrock, Castellini, Davis, & Sinnett, 1980; Thompson & Fedak, 2001). While dive durations may be extended using anaerobic glycolysis, the production of lactate requires additional post‐dive surface recuperation time and is generally thought to be a less efficient strategy (Castellini, Davis, & Kooyman, 1988; Fedak & Thompson, 1993; Kooyman et al., 1980). Yet, there are instances where marine mammals will frequently exceed aerobic thresholds. For example, benthic foraging otariid species tend to exceed their aerobic dive limit (ADL) more often than pelagic foraging species of similar size (Chilvers, Wilkinson, Duignan, & Gemmell, 2006; Costa, Gales, & Goebel, 2001; Costa, Kuhn, Weise, Shaffer, & Arnould, 2004), and the great depths to which beaked whales forage requires that they routinely dive beyond their ADL (Tyack, Johnson, Soto, Sturlese, & Madsen, 2006; Zimmer & Tyack, 2007). Additionally, southern elephant seals (*Mirounga leonina*) dive significantly longer over their post‐molt (gestational) foraging period as compared with the post‐breeding foraging trip, routinely exceeding aerobic thresholds (Hindell, Slip, Burton, & Bryden, 1992). Thus, it may be energetically beneficial for animals to exceed their ADL in order to exploit rich prey patches if acquisition of these resources outweighs the costs of longer post‐dive recovery times (Houston & Carbone, 1992).

The Weddell seal (*Leptonychotes weddellii*) offers a unique model to assess whether dive behavior differs in measurable ways between females that successfully give birth and those that do not. This is because female Weddell seals weigh the same and have the same body composition (i.e., lipid stored for energetic capital) after their winter (gestational) foraging period, regardless of whether or not they produce a pup the following year. The only difference in tissue and energy accretion during this foraging period comes from fetal growth (i.e., energetic demands of gestating females are ~13% higher than non‐reproductive seals to support a growing conceptus; Shero, Krotz, Costa, Avery, & Burns, 2015), and therefore, any differences in diving efforts over the austral winter can be attributed to gestational costs. Further, the ADL was originally defined in Weddell seals in McMurdo Sound. This was followed by decades of experiments that have shown that calculating aerobic thresholds using O_2_‐storage proteins in Weddell seals provides a reliable estimate of the ADL as determined by rises in blood lactate (Costa & Sinervo, 2004; Kooyman et al., 1980).

**Figure 1:**
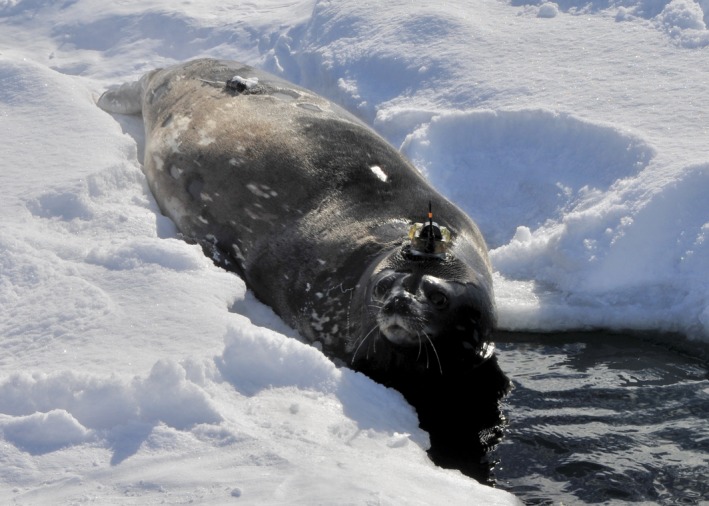
A female Weddell seal (*Leptonychotes weddellii*) outfitted with a satellite relay data logger to collect overwinter dive behavior in the Ross Sea. Photograph credit: Daniel Costa

This study tests whether foraging patterns differ between females that return with or without a pup the following year. In particular, we assess whether foraging effort is elevated during the embryo implantation period (just after the annual pelage molt) when animals are relatively lean, during the mid‐winter period, or just prior to parturition when gestational costs are greatest. This was done by comparing the over winter diving pattern of female Weddell seals (Figure 1) that successfully carried a pregnancy to term (i.e., live birth) versus those that did not give birth. The majority of animals in both reproductive groups traveled ~500 km through the Ross Sea to the continental shelf break (Goetz, 2015), and therefore, we focused on the comparison of traditional metrics of foraging effort and success (dive duration, depth, bottom time at >80% the maximum depth of a dive, and dive bouts) using dive recorders and biologging devices, and we have also developed new proxies of foraging effort. Moreover, we examined whether pregnant females exceed their calculated aerobic dive limit (cADL) or depress their DMR to support longer aerobic dives, which could then facilitate increased prey capture and higher rates of mass gain. Understanding the relationship between physiology and behavior in a top, marine predator will provide insight to their resilience to environmental perturbations, such that energy intake could still sustain reproduction. The utility of biologging devices to identify behavioral shifts that reliably predict successful reproductive events in wild animals would have broad applicability.

## 2 MATERIALS AND METHODS

### 2.1 Animal handling

Twenty‐three post‐molt adult female Weddell seals were captured on the fast‐ice along the McMurdo Sound region, Antarctica in Erebus Bay (~77°S, 165°E) and the Victoria Land coastline (~76°S, 162°E) in January/February (austral fall) 2010–2012. All the post‐molt females in this study were assumed to have not given birth earlier in the year, based on demographic records and molt status (Burns, Shero, Costa, Testa, & Rotella, 2013; R. S. Beltran & J. M. Burns, unpublished data). Animals were sedated with an initial intramuscular dose of approximately 1.0 mg/kg tiletamine/zolazepam HCl. Following a 10‐ to 15‐min induction period, animals were captured via hoop net and additional intravenous injections of ketamine and diazepam (~0.2 mg/kg and 0.012 mg/kg) were administered approximately every 10 min, or as necessary, to keep animals sedated while remaining eupneic. Pregnancy status at the time of initial handling was not determined. Reproductive outcomes were determined based on visual resightings of females with pups the next spring.

All post‐molt females were outfitted with Conductivity Temperature Depth‐Satellite Relay Dive Loggers (CTD‐SRDLs) weighing 600 g from the Sea Mammal Research Unit (University of St. Andrews, St. Andrews, Scotland) and a VHF transmitter. CTD‐SRDL tags were attached to the fur on the animals’ heads using 5 min epoxy (Loctite® or Devcon®). Data were transmitted as compressed dives (Fedak, Lovell, McConnell, & Hunter, 2002) to the Collecte Localisation Satellites, Advanced Research and Global Observation Satellite System (CLS ARGOS). Twenty females were recaptured the following spring, but three had tag failures early in the deployment. Three more animals were seen (i.e., known pregnancy outcomes) and dive records transmitted, but could not be handled due to logistical constraints. In total, dive records were obtained for 12 females that were known to have given birth (“reproductive”) the following year (*t* + 1), and for eight returning females did not produce a pup (“non‐reproductive”). Reproductive females were handled an average of 7.3 ± 1.5 days post‐partum. Of recaptured females, only 10 were still carrying their tags when recaptured. Instruments were physically recovered from these ten returning animals and contained complete dive records (i.e., no ARGOS transmission loss). Transmitted data from the remaining tags were used when recovered data were not available (Table 1).

**Table 1:** Tag deployment basics and physiological measures of Weddell seal body composition and aerobic capacities over winter (mean ± 95% CI)

		Non‐reproductive	Reproductive
Tag deployment			
Number of instruments	Recovered	5	5
	Transmitted	3	7
Duration of dive record	Recovered	278.0 ± 5.0	253.4 ± 11.9
	Transmitted	184.3 ± 127.2	209.3 ± 40.7
Number of dives	Recovered^a^	17,734 ± 2,438	15,148 ± 2,178
	Transmitted^b^	2,438 ± 1,121	3,681 ± 2,160
Physiological measures			
Mass (kg)	Fall (Post‐Molt)^a^	332.0 ± 64.1	377.9 ± 16.8
	Spring (Pre‐Breeding)^b^	405.8 ± 72.3	410.6 ± 34.7
Lipid (% Mass)	Fall (Post‐Molt)^a^	32.0 ± 2.46	31.5 ± 1.64
	Spring (Pre‐Breeding)^b^	35.8 ± 2.40	35.8 ± 1.94
TBO_2_ (ml kg lean mass^−1^)	Fall (Post‐Molt)	116.8 ± 10.8	121.6 ± 10.6
	Spring (Pre‐Breeding)	119.5 ± 5.57	113.8 ± 5.37
cADL (min)	Fall (Post‐Molt)	18.6 ± 1.52	20.3 ± 1.55
	Spring (Pre‐Breeding)	19.1 ± 1.85	18.2 ± 0.86

Note

Different superscript letters indicate that recovered tags yielded a significantly greater number of dives or a significant effect of season, respectively. There were no differences in physiology by reproductive status. Not all physiological measures could be collected at tag recovery (physiological measures, Spring: non‐reproductive *n* = 4; reproductive *n* = 9 [except for TBO_2_ and cADL where *n* = 8]). More detail on overwinter physiology in Shero, Costa, et al. (2015) and Shero, Krotz, et al. (2015).

To relate dive behavior to physiological condition, animals were weighed using a sling, tripod, and scale (MSI‐7200‐IT Dyna‐Link digital dynamometer, capacity 1,000 ± 1.0 kg) at each handling (Table 1). Body composition (%lipid) was measured using tritiated water dilution (Shero, Pearson, Costa, & Burns, 2014) and blood and muscle O_2_ stores were measured to calculate an aerobic dive limit (cADL) for each animal (Shero, Costa, & Burns, 2015).

### 2.2 Dive processing

For both recovered and transmitted records, a dive was defined as an underwater event that lasted for 4× the sampling interval and depth resolution of data loggers, or >16 s in duration and >12 m (but <2,000 m) in depth. Only dives with vertical travel speeds <5 m/s were retained, and additional outliers were detected visually using dive depth versus duration plots and discarded.

Records of individual dives contained four main inflection points where the largest change in trajectory occurred for each individual dive (Fedak et al., 2002). Bottom time, foraging efficiency, and dive shapes were determined by interpolating 100 evenly distributed mid‐depths between the four major inflection points transmitted per dive (i.e., at each 1% mid‐depth of the dive profile). Each interpolated point at >80% the maximum depth for a given dive was considered to be “bottom” time. Foraging efficiency was then calculated as (Ydenberg & Clark, 1989):

(1) $$\text{Foraging Efficiency}\;\left(\%\right)=\frac{\text{Bottom Time}}{\left(\text{Dive Duration}+\text{Post ‐ Dive Surface Duration}\right)}.$$

Dive shapes were identified by first calculating 10 mean mid‐depths per dive, and then using *K*‐means cluster analyses and *R* ^2^ and pseudo *F* statistics to identify the number of unique clusters (Schreer, Kovacs, & O'Hara Hines, 2001; Schreer, O'Hara Hines, & Kovacs, 1998). Additionally, dive descent and ascent rates were calculated as the vertical meters traveled per second from the initiation of a dive to the first inflection point, and from the last inflection point to the termination of a dive, respectively (Biuw, McConnell, Bradshaw, Burton, & Fedak, 2003).

The tags provided a separate record of animal activity budgets (%time spent diving, at surface, and hauled‐out), and the number of dives was aggregated in four, 6‐hr intervals each day. Daily activity budgets were determined by averaging activity budgets for the full‐day, and summation of dive frequencies, when all data were available for the full 24‐hr period. Days with <24‐hr data retrieval from transmitted records were excluded from analyses to avoid biases from diurnal foraging patterns (Boehme et al., 2016).

### 2.3 Aerobic capacity and physiological constraint to diving

To better understand how aerobic capacity may constrain dive behavior across the austral winter, dive duration was first compared to the calculated aerobic dive limit (cADL), determined as total body oxygen (TBO_2_) stores divided by an average diving metabolic rate (DMR) of 1.6 × Kleiber (Kleiber, 1975; Shero, Costa, et al., 2015; Williams, Fuiman, Horning, & Davis, 2004), for each individual. A linear interpolation was used to transition from the TBO_2_ and total body mass measured at the start of the winter tag deployment, to physiological measures the following spring, when measured at both time points (non‐reproductive: *n* = 4, reproductive: *n* = 8). Fetal mass was calculated using a Gompertz embryo growth curve fit to Weddell seal fetal mass from Smith (1966), with the assumptions that active gestation lasts 10 months (M. R. Shero, G. P. Adams, R. B. McCorkell, A. L. Kirkham, & J. M. Burns, unpublished data) and pup birth mass is 27 kg (Wheatley et al., 2006). Fetal mass was then added to reproductive female total body mass. For each day during the tag deployment, estimated TBO_2_ and body mass from linear interpolations were used to calculate a cADL, and each dive was categorized as either being greater or less than the cADL.

A second approach to determine physiological constraints on dive behavior assumed that the vast majority of all dives were aerobic (Kooyman et al., 1980; Kooyman, Castellini, Davis, & Maue, 1983) and estimated the diving metabolic rate from dive durations and calculated TBO_2_ stores. In this approach, the behavioral ADL (bADL) for each individual was determined as the 95th percentile of dive durations each day, for those days with *n* ≥ 20 dives (Burns & Castellini, 1996; Kooyman et al., 1983). The DMR that would be necessary to support this dive duration aerobically given the animals’ TBO_2_ stores was calculated as:

(2) $$\text{Calculated Diving Metabolic Rate}\left(\text{mg}\cdot {\text{kg}}^{-1}\cdot {\text{min}}^{-1}\right)=\frac{{\text{TBO}}_{2}\text{stores}\left(\text{ml}\cdot {\text{kg}}^{-1}\right)}{\text{bADL}\left(\text{min}\right)}.$$

### 2.4 Characterization of overwinter dive bouts

To assess organization of larger foraging effort, dives were grouped into bouts for the 10 animals with entire overwinter dive records from recovered tags (non‐reproductive *n = *5; reproductive *n = *5). This could only be done for complete archived dive records downloaded from the recovered tags, as missing dives (present in the transmitted dive records) would concatenate surface intervals and artificially shorten bouts. To evaluate the number of processes that best captured the structuring of bouts, a two‐process non‐linear least squares model assuming a Poisson distribution was first fit to log frequency plots of post‐dive surface intervals for each animal. The break point was used to identify bout‐ending criterion (BEC) to divide surface gaps into short (within bout) and long processes (between bouts; Slater & Lester, 1982; Feldkamp, DeLong, & Antonelis, 1989; Sibly, Nott, & Fletcher, 1990; Berdoy, 1993). Then, three‐process models were fit to post‐dive surface intervals, identifying BEC1 (post‐dive surface intervals less than BEC1 grouped the dives into short bouts; BOUT_short_) and BEC2 (which nested short bouts within longer bouts; BOUT_long_). All models were fit using the “diveMove” package in R, following methods outlined in Sibly et al. (1990) and Berdoy (1993). To assess whether behaviors were better captured using both BOUT_short_ and BOUT_long_, two‐ versus three‐process model fit was compared using AICc and ANOVA *F*‐tests. A BOUT_short_ and BOUT_long_ was considered to be >5 dives made within BEC1 and BEC2, respectively, and bout characteristics (i.e., duration, number of dives, number of BOUT_short_ within BOUT_long_) were assessed across the year and between reproductive groups.

To characterize bout types based on the frequency and depth of dives within them, bout shapes were also identified. Bout shapes were determined in a similar fashion to shapes of individual dives; however, the maximum depth of each dive within the pre‐defined bout was used as the initial points defining the bout “shape” and 100 midpoints were interpolated to create shape clusters as with individual dives. Finally, bout efficiency was calculated as:

(3) $$\text{Bout Efficiency}\left(\%\right)=\frac{\text{Dive Time at}>80\%\text{Maximum Bout Depth}}{\left(\text{Bout Duration}+\text{Post ‐ Bout Surface Interval}\right)}.$$

The proportion of total dives that occurred within bouts was determined, and the last dive in a bout was compared to all dives within the bout to determine whether bouts ended after a dive that exceeded physiological capacity.

### 2.5 Statistical analyses

Generalized additive mixed models (GAMMs with the “mgcv” package in R v. 3.2.4) were used to determine how dive efforts, activity budgets, dive and bout types, and aerobic thresholds (i.e., the probability of exceeding the cADL and the calculated DMR) changed across the year and in response to reproductive status. Julian day was used as a smoother in models, and reproductive status in year *t* + 1 was a factor (with and without a reproductive status × Julian day interactive effect) with a penalized thin‐plate regression spline. Animal ID was included as a random effect and a corAR1 temporal autocorrelation term was included. The best‐fit model was identified using Akaike information criterion tests corrected for small sample size (AICc) in the R “MuMIn” package (Zuur, Saveliev, & Ieno, 2014). Because adding the interactive term (reproductive status × Julian day) frequently yielded the better fit model as determined by AICc, dive metrics were also compared between reproductive groups within each month to provide more detailed temporal comparisons. Models were validated to ensure there was not overdispersion or heterogeneity of residuals. All results are presented as the mean ± 95% confidence interval; trends are presented (*α* = 0.10) and significance was set at the *α* = 0.05 level.

## 3 RESULTS

### 3.1 Overwinter physiology: body composition and aerobic capacity

**Figure 2:**
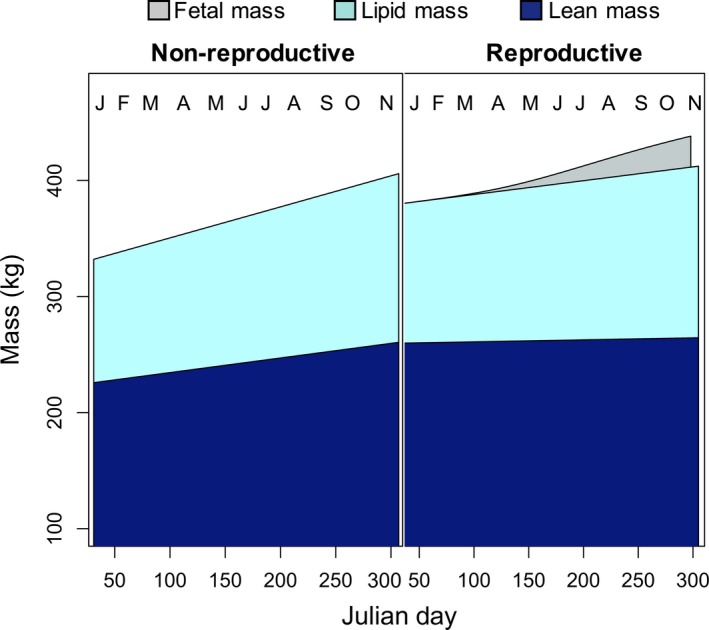
Fetal mass was the only difference in mean body mass and energy gain between females that returned the following year with (reproductive, *right*) and without a pup (non‐reproductive, *left*)

The proportion of females returning in year *t* + 1 that gave birth in this study was similar to that of the overall population (Chambert, Rotella, Higgs, & Garrott, 2013; Hadley et al., 2007; Hadley, Rotella, & Garrott, 2006; Proffitt et al., 2007). Both reproductive and non‐reproductive seals gained a significant amount of body mass and lipid mass across the overwinter foraging period (Figure 2; Table 1; *P*s <0.001; Shero, Krotz, et al., 2015), and there were no differences in body composition by reproductive status. Thus, the only difference between reproductive groups in energy requirements over the winter was the additional energy that reproductive females had to allocate in support of fetal tissue deposition and the heat increment of gestation (Figure 2; Table 1). Mass‐specific TBO_2_ stores and the cADL were similar across seasons and reproductive status (Shero, Costa, et al., 2015; Shero, Krotz, et al., 2015; Table 1).

### 3.2 Overwinter foraging between reproductive groups

**Figure 3:**
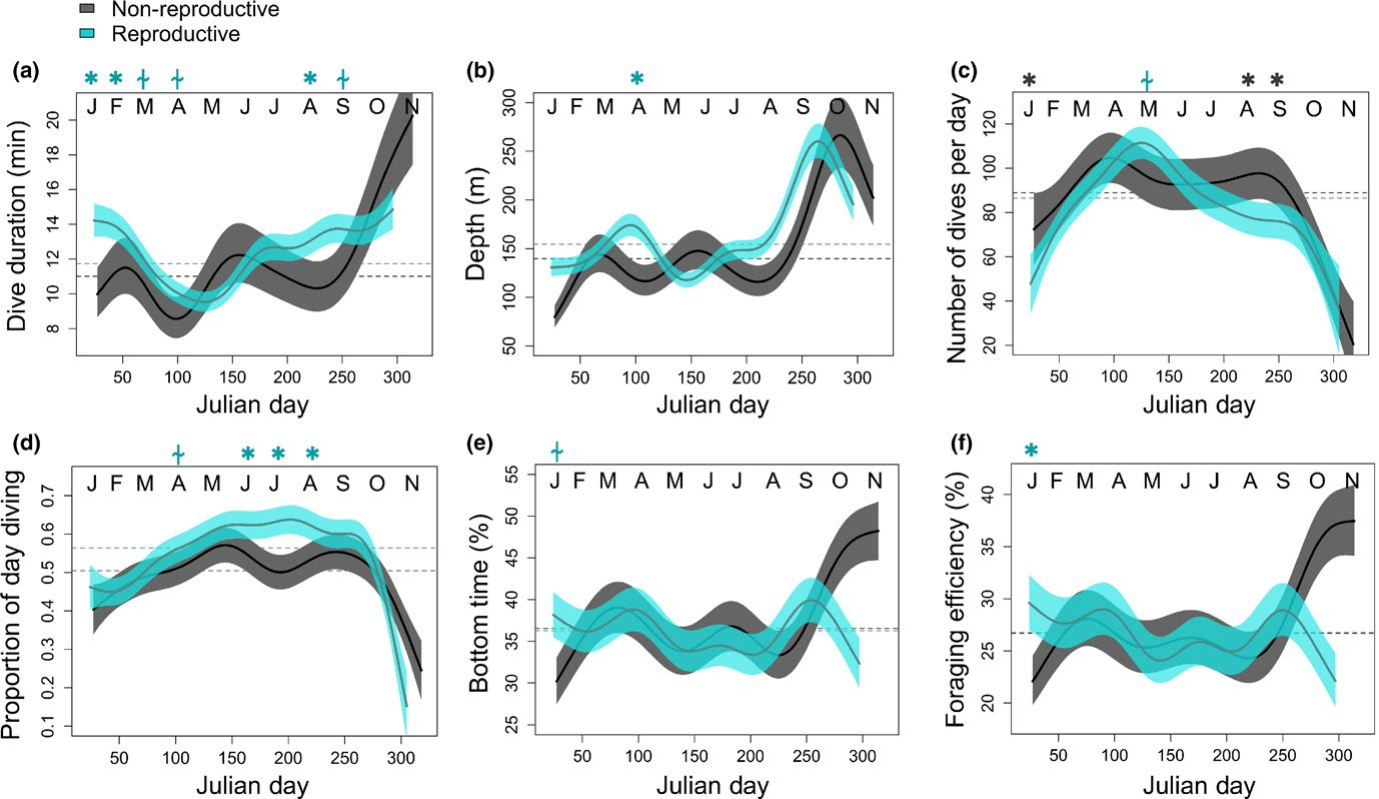
Generalized additive mixed models showing trends in (a) dive duration, (b) depth, (c) number of dives made per day, (d) the proportion of each day spent diving, (e) bottom time (as a percentage of total dive duration), and (f) foraging efficiency across the year (±95% CI). Julian day had a significant effect on all dive parameters. Reproductive groups are shown as: *gray* = non‐reproductive; *blue* = reproductive. Month is abbreviated at the top of each panel and *Asterisk* = significant differences between reproductive groups for a given month (*p* < 0.05); “⍭” = trend (*p* < 0.10). Symbol color corresponds to the reproductive group with the greater dive measure; dashed horizontal lines represent means across the tag deployment

As the winter season progressed, differences in diving behavior between reproductive and non‐reproductive females began to emerge. These differences were superimposed on strong seasonal changes in behavior. All seals increased dive durations and depth (Figure 3a,b; all behaviors ~Julian day, *p* < 0.001) mid‐winter, from ~May until the next breeding period in October. During those months when animals made longer and deeper dives, they also made fewer dives per day (Figure 3c). Dive frequencies and daily dive activity increased directly post‐molt from January until April (Figure 3c‐d). There was a sharp decline just prior to the breeding season, as dives became longer and deeper.

Gestating seals tended to behave in ways suggestive of increased dive efforts relative to non‐reproductive seals across the austral winter. For example, females that returned the next year with a pup made significantly longer dive durations immediately post‐molt (Figure 3a; January: *F* = 9.4, *p* = 0.002; February: *F* = 6.9, *p* = 0.009) and mid‐winter (August: *F* = 4.7, *p* = 0.030). Reproductive females also made deeper dives throughout the winter (Figure 3b; entire deployment *F = *3.9, *p* = 0.048). While reproductive females tended to make fewer dives each day (January: *F* = 7.9, *p* = 0.007; August: *F* = 16.2, *p* < 0.001; September: *F* = 4.1, *p* = 0.043), reproductive females still spent a significantly larger proportion of the day diving during mid‐winter (Figure 3c,d; June: *F* = 5.8, *p* = 0.017; July: *F* = 7.8, *p* = 0.005; August: *F* = 6.2, *p* = 0.013), due to their longer dive durations. Dive depth, frequency, and daily dive time declined dramatically in all females in October, as the animals returned to the breeding colonies. However, non‐reproductive females increased their dive durations from September to November, while reproductive females did not.

Bottom time (minutes and percent) and foraging efficiency were relatively high in all females following the molt (January‐March) coincident with shallower dives (Figure 3e–f). Reproductive females had significantly greater bottom time (minutes) and foraging efficiency than non‐reproductive females at the start of winter foraging (Bottom time—January: *F* = 6.4, *p* = 0.012; February: *F* = 5.2, *p* = 0.023; Foraging Efficiency—January: *F* = 7.5, *p* = 0.006). Bottom time and foraging efficiency declined mid‐winter (May‐September) in both reproductive groups. However, when dives were significantly deeper toward the end of the winter foraging period (August–September), bottom time and foraging efficiency also increased in both reproductive groups (Figure 3e–f).

**Figure 4:**
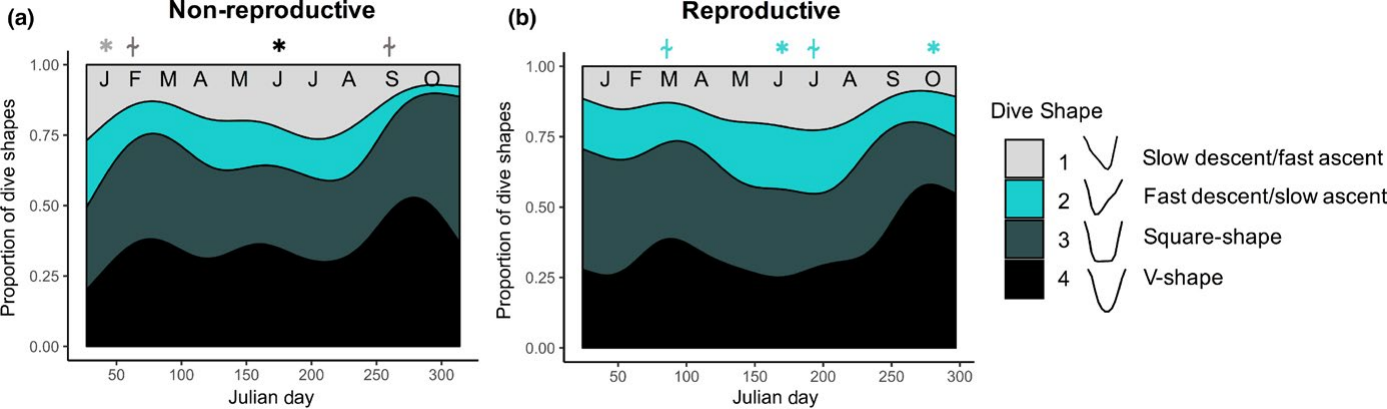
Generalized additive mixed models showing the proportion of individual dive shapes for (a) non‐reproductive and (b) reproductive female Weddell seals across the year. All dive shapes exhibited significant relationships with Julian day. Month is abbreviated at the top of each panel and *Asterisk* = significant differences between reproductive groups for a given month (*p* < 0.05); “⍭” = trend (*p* < 0.10). The symbol is located above the reproductive group that had the greater dive frequency, and the symbol is color coded to correspond with the class of dive

Four dive shapes were identified, with the majority of dives being long and deep‐square and V‐shape, indicative of efficient foraging (Table 2). The proportion of dives that fell into the deep‐square‐shape and V‐shape categories were highest at the start of the winter foraging period, and again late winter (Figure 4). Reproductive females tended to make more fast‐descent/slow‐ascent dives (June: *F* = 4.7, *p* = 0.030; October: *F* = 25.2, *p* < 0.001), whereas non‐reproductive females made more slow‐descent/fast‐ascent dives at the start of winter foraging (January: *F* = 8.7, *p* = 0.003).

**Table 2:** Characteristics (weighted mean ± 95% CI) of Weddell seal dive shapes as determined by cluster analysis after interpolating mid‐depths

Dive parameter	Type 1 Slow descent/fast ascent	2 Fast descent/slow ascent	3 Square‐shape	4 V‐shape
Frequency of dive type (%)	16.9 ± 1.28^a^	16.1 ± 1.92^a^	34.2 ± 3.20^b^	32.7 ± 1.76^b^
Dive duration (min)	10.3 ± 0.83^a^	12.3 ± 0.48^b^	13.6 ± 0.80^c^	10.1 ± 0.58^d^
Post‐dive surface duration (min)	2.73 ± 0.21^a^	3.07 ± 0.18^b^	3.22 ± 0.17^c^	3.19 ± 0.20^d^
Maximum depth (m)	83.1 ± 6.14^a^	118.7 ± 8.29^b^	154.7 ± 12.3^c^	176.9 ± 14.8^d^
Bottom time (min; >80% max dive depth)	1.96 ± 0.13^a^	2.17 ± 0.11^b^	7.26 ± 0.62^c^	3.84 ± 0.27^d^
Bottom time (%Dive)	21.2 ± 0.56^a^	18.6 ± 0.51^b^	51.8 ± 1.52^c^	36.5 ± 0.56^d^
Ascent rate (m/s)	0.78 ± 0.03^a^	0.37 ± 0.02^b^	0.93 ± 0.03^c^	0.84 ± 0.05^d^
Descent rate (m/s)	0.45 ± 0.03^a^	0.97 ± 0.08^b^	1.13 ± 0.05^c^	1.08 ± 0.06^c^
Foraging efficiency (%)	15.6 ± 0.42^a^	13.9 ± 0.34^b^	40.2 ± 1.50^c^	25.3 ± 0.77^d^
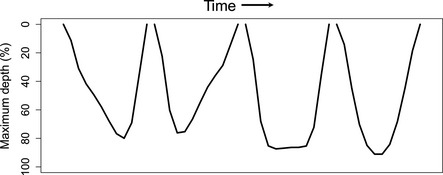				

Note

Different superscript letters indicate significant differences between dive shape clusters.

### 3.3 Aerobic capacity and dive behavior

**Figure 5:**
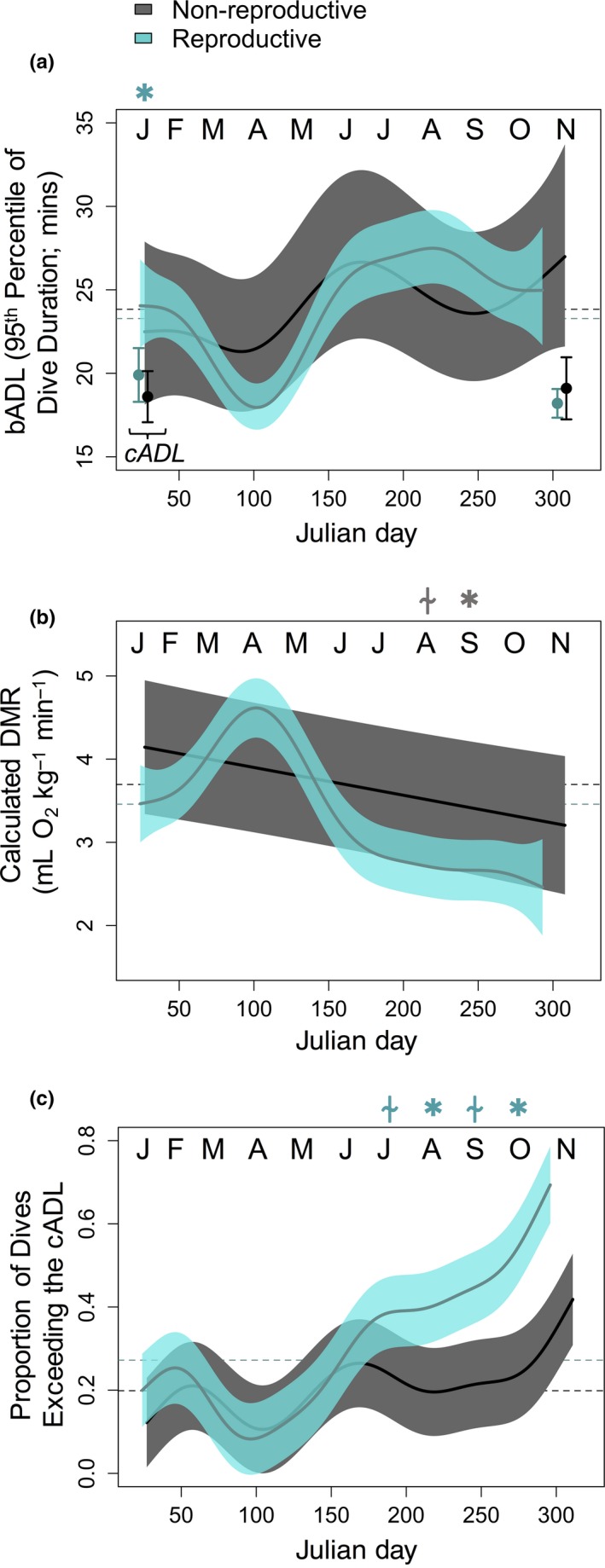
(a) The behavioral aerobic dive limit (bADL) differed across the winter (points = the cADL is shown for comparison), and (b) was used to calculate the Diving Metabolic Rate (DMR) needed to support dive durations. (c) The proportion of dives exceeding the calculated aerobic dive limit (cADL) across the austral winter (±95% CI). Reproductive groups are shown as: gray = non‐reproductive; blue = reproductive. Month is abbreviated at the top of each panel and Asterisk = significant differences between reproductive groups for a given month (*p* < 0.05); “⍭” = trend (*p* < 0.10). Symbol color corresponds to the reproductive group with the greater dive measure; dashed horizontal lines represent means across the tag deployment

Because animals did not increase O_2_ stores to support longer overwinter dives, they must have either depressed DMRs to lengthen their aerobic dive window, or increased how often dives exceeded the cADL across the winter foraging period. Throughout the entire study, the bADL was always higher than the cADL (Figure 5a), and reproductive females had a significantly longer bADL than non‐reproductive females at the start of winter foraging (January: *F* = 6.5, *p* = 0.016). The calculated DMR that would be necessary to support the reproductive female's long dives tended to be lower than non‐reproductive females (Figure 5b; by 9.87% ± 1.74% on average for the entire deployment). However, in late winter, when reproductive females were making long dives, their longer bADL suggested that their DMR was 22.1% ± 0.25% lower than that of non‐reproductive females (September: *F* = 4.3, *p* = 0.039).

**Figure 6:**
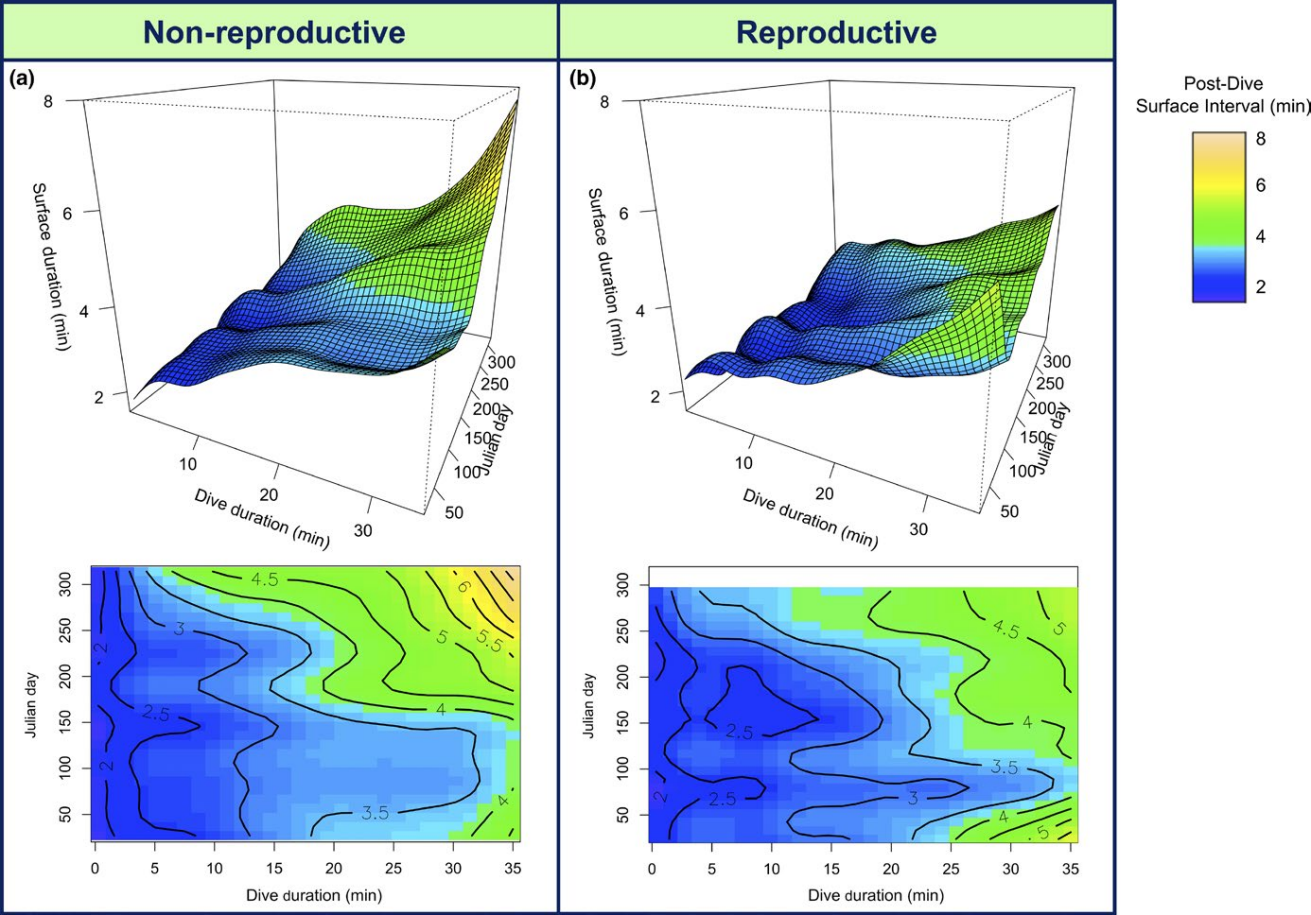
(*Top*) three‐dimensional GAMMs showing the relationship between dive duration and post‐dive surface duration changes throughout the year in both (a) non‐reproductive and (b) reproductive female Weddell seals. (*Bottom*) contour values show post‐dive surface duration. Note that dive duration was limited to <35 min due to low sample size of longer dives

Conversely, animals may have exceeded their cADL more often to achieve longer dives. If this were the case, all animals would have exceeded their cADL more often in the January‐February post‐molt period, and just prior to the breeding season the next October, as compared to the mid‐winter period (Figure 5c). In spring, reproductive females exceeded the cADL significantly more often than non‐reproductive seals (Figure 5c; August: *F* = 4.1, *p* = 0.044; October: *F* = 6.9, *p* = 0.009). Exceeding aerobic thresholds necessitates longer surface recuperation times, and indeed, the relationship between dive duration and post‐dive surface time changed across the year in both reproductive groups (surface time ~dive duration × Julian day interactive term: non‐reproductive: *F* = 36.1, *p* < 0.001; reproductive: *F* = 36.8, *p* < 0.001). Thus, for a given dive duration, surface recuperation time was higher mid‐winter and just prior to the next breeding season, regardless of reproductive status, suggesting animals were making more anaerobic dives (Figure 6).

### 3.4 BOUT organization

**Figure 7:**
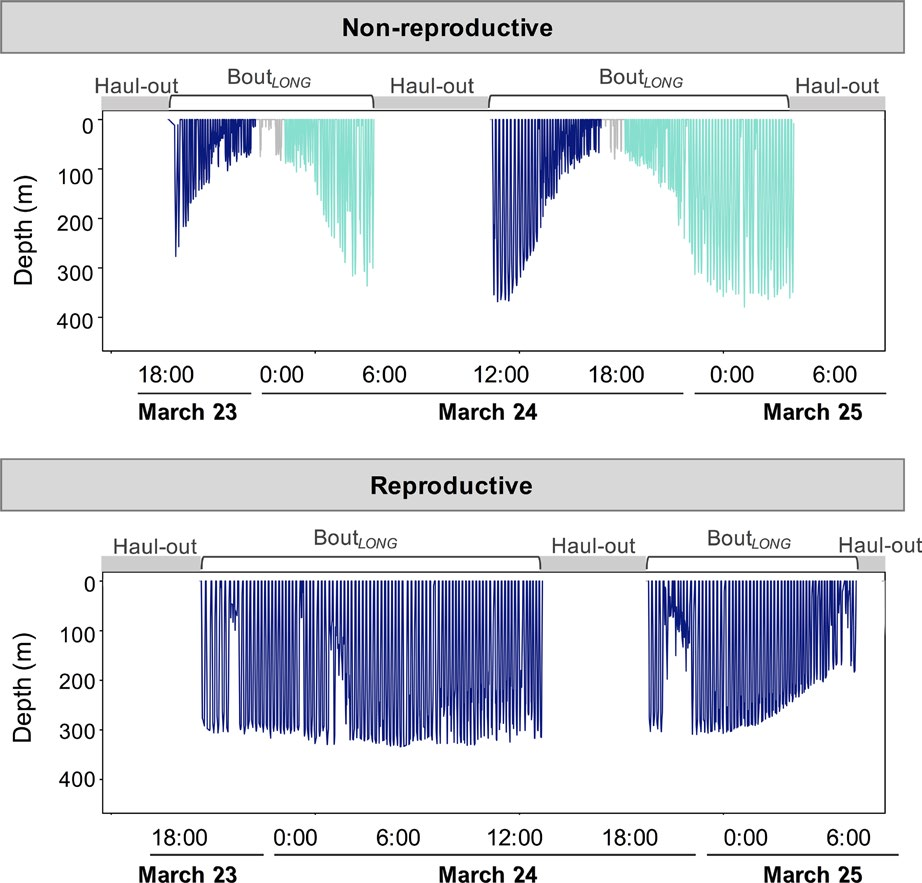
Examples of bout composition between a non‐reproductive and reproductive female. The non‐reproductive females had significantly more BOUT_short_ nested within the longer daily foraging effort. (*Top panel*) Each daily BOUT_long_ contained two BOUT_short_ (blue and green, respectively) with a few (<5) dives inbetween that had post‐dive surface intervals that exceeded BEC1. The longer post‐dive surface intervals were associated with dramatic changes in dive depths. (*Bottom panel*) this is in contrast to reproductive females that frequently only made one large bout per day (i.e., BOUT_short_ = BOUT_long_) without taking a rest period that exceeded BEC1

Dives were further classified into bouts (Figure 7), and three‐process models provided significantly better fit to log frequency post‐dive surface interval plots, as compared with two‐process models (Table 3). Only 0.8% of dives were not included in BOUT_short_ or longer trips to sea (BOUT_long_). The vast majority of dives (93.8% ± 1.2%) were performed in bouts consisting of >5 successive dives that ended after the post‐dive surface interval reached 10.35–21.71 min (BOUT_short_), depending on the bout‐ending criterion determined from three‐process models for each individual. These short bouts were then organized into longer bouts that ended after the post‐dive surface interval exceeded 50.23–111.68 min (BOUT_long_). Because long bouts were a series of short bouts, the dives within them were not analyzed separately. The bout‐ending criteria (post‐dive surface intervals between processes; BEC1 and BEC2) did not differ between reproductive groups (BEC1: *t*_8.0_ = −0.1, *p* = 0.966; BEC2: *t*_7.7_ = −1.2, *p* = 0.275).

**Table 3:** Resulting bout‐ending criteria (BEC) values for two‐ versus three‐process models; figure shows BEC determination for one individual as an example

Animal ID	Reproductive status	Two‐process model		Three‐process model			Comparison of models		
		BEC (min)	AICc	BEC1 (min)	BEC2 (min)	AICc	ΔAICc	ANOVA	
WS10‐03	Non‐Reproductive	33.93	2,309.2	12.43	62.42	2,117.5	−191.7	*F*_2, 176.3_ = 109.9	*p* < 0.001
WS10‐05	Non‐Reproductive	46.76	2,429.3	13.85	77.28	2,255.4	−173.9	*F*_2, 136.7_ = 97.7	*p* < 0.001
WS10‐07	Non‐Reproductive	32.70	2,345.3	14.72	71.29	2,197.8	−147.5	*F*_2, 139.7_ = 82.6	*p* < 0.001
WS12‐04	Non‐Reproductive	38.97	2,338.1	21.62	111.68	2,147.0	−191.1	*F*_2, 161.4_ = 109.0	*p* < 0.001
WS12‐13	Non‐Reproductive	33.73	2,054.6	20.63	92.27	1,892.9	−161.7	*F*_2, 126.7_ = 91.8	*p* < 0.001
WS10‐01	Reproductive	22.55	1,682.7	10.35	55.11	1,526.8	−155.9	*F*_2, 153.7_ = 91.4	*p* < 0.001
WS10‐02	Reproductive	26.87	2,181.5	17.53	50.23	2,111.0	−70.5	*F*_2, 62.9_ = 38.8	*p* < 0.001
WS12‐01	Reproductive	29.78	1,578.9	21.71	81.69	1,508.4	−70.5	*F*_2, 57.3_ = 39.3	*p* < 0.001
WS12‐09	Reproductive	30.89	2,251.9	16.58	80.74	2,147.9	−104.0	*F*_2, 108.6_ = 57.6	*p* < 0.001
WS12‐12	Reproductive	29.80	1,890.8	16.50	81.48	1,751.7	−139.1	*F*_2, 127.0_ = 79.1	*p* < 0.001
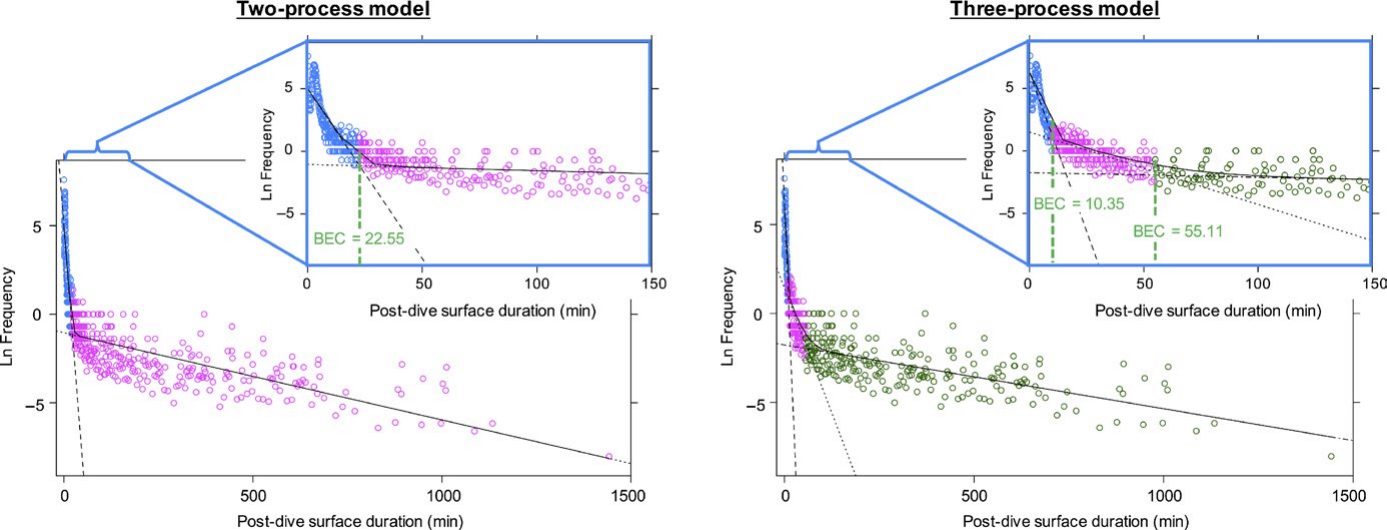									

Note

Akaike information criteria and ANOVA tests were used to assess whether adding a third process significantly improved model fit. In all Weddell seals, adding the third process improved models.

**Figure 8:**
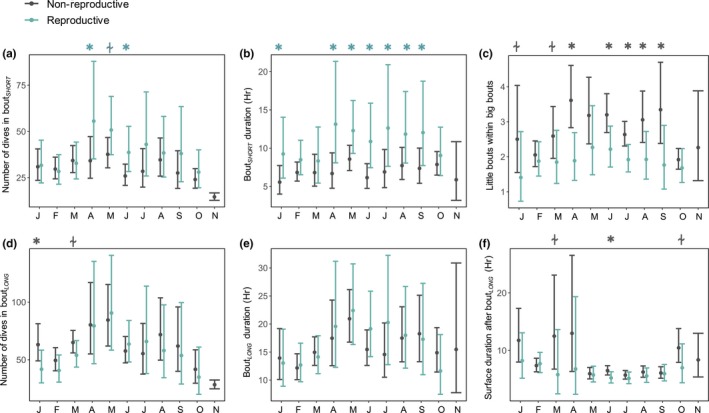
Generalized additive mixed models showing how the (a,b) number of dives and bout duration changed across the year in BOUT_short_. (c) The number of BOUT_short_ nested within BOUT_long_ differed across the year and by reproductive group. Daily foraging efforts were organized into BOUT_long_, and (d,e) the number of dives and duration of BOUT_long_ increased mid‐winter, (f) while surface duration exhibited an inverse relationship with bout durations (±95% CI). Reproductive groups are shown as: *gray* = non‐reproductive; *blue* = reproductive. Month is abbreviated at the bottom of each panel and *Asterisk* = significant differences between reproductive groups for a given month (*p < *0.05); “⍭” = trend (*p* < 0.10). Symbol color corresponds to the reproductive group with the greater dive measure

The foraging bouts of female Weddell seals differed between females that successfully produced a pup, and those that did not. Reproductive females tended to make a greater number of dives per BOUT_short_ during the winter (Figure 8a; *overall F* = 3.2, *p* = 0.074; specifically in April: *F* = 4.3, *p* = 0.038; June: *F = *6.3, *p* = 0.012), and because dives on average were longer (*see above*) reproductive females had significantly longer BOUT_short_ durations than non‐reproductive females (Figure 8b; reproductive: 10.9 ± 2.84 hr; non‐reproductive: 7.06 ± 1.29 hr; *overall: F = *10.4, *p* = 0.001; January, April–September: *all F* > 4.5, *p* < 0.05). Reproductive females increased BOUT_short_ durations mid‐winter (*F* = 10.2, *p* < 0.001), whereas there were no temporal changes in non‐reproductive female BOUT_short_ durations (*F* = 2.5, *p* = 0.111). Non‐reproductive females made significantly more BOUT_short_ within BOUT_long_ as compared to reproductive females (Figure 8c; *overall: F* = 19.6, *p* < 0.001; April, June–September: *all F* > 6, *p < *0.05). As a result, reproductive and non‐reproductive females had a similar number of dives within BOUT_long_, and BOUT_long_ durations (16.8 ± 2.0 hr; Figure 8d,e). Surface intervals between BOUT_long_'s exhibited an inverse relationship with bout duration, and non‐reproductive females had significantly longer rest periods between long bouts (Figure 8f; *overall F* = 6.5, *p* = 0.011; specifically, June: *F* = 5.3, *p* = 0.022). In both reproductive groups, the last dives in BOUT_short_ were significantly shorter in duration (8.5 ± 1.0 vs. 11.4 ± 1.1 min; *F* = 1,275, *p* < 0.001) and also shallower (105.9 ± 1.0 vs. 153.8 ± 1.1 m; *F* = 1,290, *p* < 0.001), as compared with the preceding dives in the bout.

**Figure 9:**
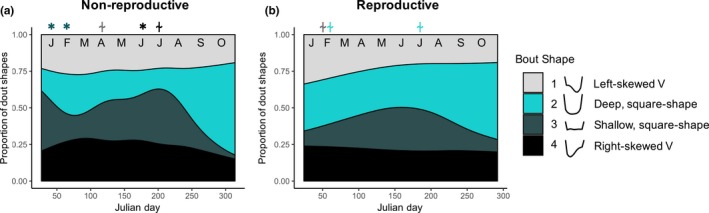
Generalized additive mixed models showing the proportion of bout shapes for (a) non‐reproductive and (b) reproductive female Weddell seals across the year. All bout shapes exhibited significant relationships with Julian day. Month is abbreviated at the top of each panel and Asterisk = significant differences between reproductive groups for a given month (*p* < 0.05); “⍭” = trend (*p* < 0.10). The symbol is located above the reproductive group that had the greater bout frequency, and the symbol is color coded to correspond with the class of bout

Cluster analyses were only conducted on BOUT_short_ as these contained >90% of dives and revealed four main BOUT_short_ shapes, with relatively even distributions of left‐skewed‐“V,” deep‐square, shallow‐square, and right‐skewed‐“V” shaped bouts (Table 4). Deep‐square BOUT_short_ tended to be comprised of fewer dives that were of longer duration and exceeded the cADL more often, reached greater mean depths, and had greater within‐bout dive:surface time ratios (Table 4). In both reproductive and non‐reproductive females, the frequency of deep‐square BOUT_short_ increased from mid‐winter until the next breeding season (Figure 9). Consequently, the frequency of all other BOUT_short_ types declined just prior to the spring. Reproductive females had a significantly lower proportion of shallow‐square bouts in January‐February (January: *F* = 9.7, *p* = 0.003; February: *F* = 15.18, *p* < 0.001), directly following the molt, as compared with non‐reproductive females. Similarly, reproductive females made significantly fewer right‐skewed “V” bouts than non‐reproductive females mid‐winter (June: *F* = 4.3, *p* = 0.038). There were no other significant differences in bout type frequencies between reproductive groups over winter.

**Table 4:** Characteristics (weighted mean ± 95% CI) of Weddell seal BOUT_short_ shapes as determined by cluster analysis, after interpolating maximum dive depths

Bout parameter (BOUT_short_)	Type 1 Left‐skewed V	2 Deep, Square	3 Shallow, Square	4 Right‐skewed V
Frequency (%)	23.4 ± 1.50^a^	30.0 ± 6.02^a^	23.7 ± 4.99^a^	22.9 ± 2.11^a^
Number of dives	36.0 ± 4.06^a^	25.6 ± 3.06^b^	45.2 ± 7.87^c^	33.5 ± 3.60^d^
Mean dive duration (min)	11.5 ± 0.96^a^	14.7 ± 1.28^b^	9.0 ± 0.62^c^	10.9 ± 0.93^d^
Total dive time in Bout (min)	432.8 ± 61.3^a^	391.8 ± 57.3^a,b^	427.6 ± 91.4^a^	382.1 ± 55.2^b^
Mean surface duration (min)	4.00 ± 0.34^a^	4.41 ± 0.43^b^	3.62 ± 0.30^c^	3.90 ± 0.32^a^
Total surface time in Bout (min)	124.0 ± 14.2^a^	100.9 ± 13.4^b^	144.1 ± 31.4^c^	112.8 ± 14.3^a^
Bout dive:surface ratio	3.32 ± 0.32^a^	3.86 ± 0.35^b^	2.96 ± 0.38^c^	3.27 ± 0.35^a^
Bout duration (hr)	9.42 ± 1.26^a^	8.23 ± 1.15^b^	9.69 ± 2.06^a^	8.35 ± 1.14^b^
Mean depth (m)	141.9 ± 10.9^a^	222.1 ± 23.5^b^	93.4 ± 10.1^c^	136.6 ± 10.4^a^
Max depth (m)	333.4 ± 29.3^a^	334.8 ± 32.0^a^	384.9 ± 44.2^b^	340.8 ± 27.0^a^
Dives reaching >80% Bout Max depth (%)	18.7 ± 1.07^a^	48.7 ± 1.71^b^	7.40 ± 0.61^c^	18.7 ± 1.14^a^
Post‐Bout surface (min)	195.0 ± 27.4^a^	258.3 ± 53.1^b^	229.1 ± 72.0^a^	179.2 ± 16.3^a^
Bout efficiency (%)	14.0 ± 0.77^a^	35.1 ± 1.89^b^	5.37 ± 0.40^c^	13.7 ± 0.94^a^
Dives exceeding cADL (%)	19.5 ± 5.12^a^	30.8 ± 9.01^b^	11.8 ± 3.30^c^	17.6 ± 5.07^a^
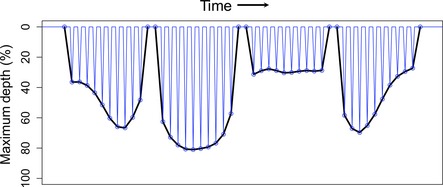				

Note

BOUT_long_ shapes were essentially identical, but consisted of a greater number of dives and were of longer duration (*n* = 10 for all parameters, except *n* = 7 for dives > cADL). Different superscript letters indicate significant differences between bout shape clusters.

## 4 DISCUSSION

This study shows that there is significant variation in dive behavior across the overwinter (gestational) foraging period in female Weddell seals. While seasonal differences may be driven by changes in prey‐fields, there is no evidence that reproductive and non‐reproductive females forage in different areas and/or on different species (Goetz, 2015; Goetz, Burns, Hückstӓdt, Shero, & Costa, 2017), so here we concentrate on the differences in behavior that are associated with putative pregnancy status. Differences in dive behavior suggest that foraging effort and energy acquisition are greater in gestating Weddell seals at the end of the annual molt (early winter). Further, the finding that reproductive females markedly increase their foraging effort during the late gestational period (late winter) suggests that pregnant Weddell seals meet the additional energetic cost of the growing fetus by increasing dive duration, depth, and the proportion of each day spent diving, and that these differences may be facilitated by a decrease in DMR.

In addition to fueling self‐maintenance costs, overwinter foraging must provide sufficient energy to support the cost of gestation. Because the only difference in overwinter mass gains between females that successfully produce a pup in year *t* + 1 and those that do not, is the mass of the fetus (Shero, Krotz, et al., 2015), differences in dive behavior between reproductive groups likely reflect the additional foraging required to supply energy to a growing fetus (Brody, 1945; Gittleman & Thompson, 1988). This study has clearly shown that the increased energetic demand of gestation is reflected at all levels of behavioral organization. There appear to be two periods when foraging differences are most apparent: immediately post‐molt, when implantation occurs and fetal growth accelerates, and in late winter, during the last trimester of pregnancy when energetic costs of gestation are highest (Brody, 1945; Gittleman & Thompson, 1988). Dive duration, bottom time, and foraging efficiency were all greater in reproductive seals directly following the molt (during the embryo implantation period) as compared to females that failed to produce a pup the next year and show that these reproductive individuals were foraging in the benthos longer or at the depth layer where preferred pelagic prey were found (“bottom,” >80% maximum dive depth). This suggests that the period directly following the annual molt may be a critical time for females to regain mass and condition (lipid) lost during the breeding season, before the onset of winter (Beck, Bowen, McMillan, & Iverson, 2003; Carlini, Marquez, Daneri, & Poljak, 1999; McDonald et al., 2008; Robinson et al., 2012) such that animals can maintain early pregnancy and prevent embryo loss (Pitcher, Calkins, & Pendleton, 1998). It is possible that some non‐reproductive females were pregnant but lost the fetus later during the austral winter. Regardless of the cause, females that did not increase diving effort just following the molt did not return with pups the next spring (Boyd, 1984; Pitcher et al., 1998).

In addition to basic dive metrics, reproductive Weddell seals organized their daily foraging activities in ways that suggested increased effort mid‐ to late winter, when compared to non‐reproductive animals. Reproductive female daily foraging efforts were structured into one long daily foraging bout; however, non‐reproductive females made several relatively short bouts interrupted by long surface intervals (i.e., more frequent BOUT_short_ nested within one long daily foraging event). Therefore, it appears that non‐reproductive seals took more breaks within their daily foraging activities, whereas reproductive seals worked harder for longer periods without rest. There have been limited studies assessing foraging costs associated with gestation in wild mammals, but findings in this study are similar to pregnant bats which will take more risks and choose to forage under challenging environmental conditions with unpredictable foraging success, as compared to males (Grinevitch, Holroyd, & Barclay, 1995). This is in contrast to other mammals that have the ability to alter their diet to minimize food processing time and thus can spend more time resting (Rose, 1994). For Weddell seals, prey processing time may also influence the structuring of daily foraging efforts. For example, the capture of large prey, such as toothfish (~70–90 kg; *Dissostichus mawsoni*) that require processing and handling prior to consumption (taking hours to complete; Fuiman, Madden, Williams, & Davis, 2007; Ponganis & Stockard, 2007), likely ends a dive bout. Alternatively, the end of a bout could be due to a physiological threshold, where CO_2_ and lactate build‐up occurs after multiple successive dives. Both are known to stimulate respiration, and animals may use these chemical cues to return to the surface to recover and repay O_2_ debts (Stephenson, 2005) before beginning the next BOUT_short_ (within 50–111 min).

Reproductive females did not have greater TBO_2_ stores that would facilitate increased dive durations and more successive dives within bouts (Shero, Costa, et al., 2015). Therefore, differences in dive duration and bout patterns between reproductive and non‐reproductive females may be driven by seasonal and pregnancy‐related changes in metabolic rate that support longer dives. While this study was unable to directly measure DMR over winter, DMRs estimated in this study from the bADL and TBO_2_ stores were consistent with direct measurements from isolated hole experiments (Williams et al., 2004). In pinnipeds, metabolic rates are elevated during the annual pelage molt (Boily, 1996; Boyd, Arnbom, & Fedak, 1993) and indeed, all Weddell seals in this study had higher calculated DMRs at the start of winter (post‐molt) foraging. Previous work suggests that pregnancy in pinnipeds is associated with hypometabolism (Renouf & Gales, 1994; Sparling, Speakman, & Fedak, 2006) and estimated DMRs in this study support the notion that pinnipeds may depress metabolic rate during gestation. Similarly, basal metabolic rate (BMR) varies across pregnancy in humans, with some individuals substantially lowering BMR below pre‐pregnancy values during the first six months of gestation, followed by elevated metabolic rates in late pregnancy (Prentice, Goldberg, Davies, Murgatroyd, & Scott, 1989). Notably, in thin women, the suppression of BMR in early pregnancy was enough to completely offset the costs of fetal growth, incurring no net energetic requirements, and thus may reflect nutritional status (Lawrence, Coward, Lawrence, Cole, & Whitehead, 1987; Prentice et al., 1989).

The increase in bADL across the winter in all seals in this study suggests that there is a significant decline in estimated mass‐specific DMR across the winter, and that this decline is more prominent in pregnant females. Lowering BMR and DMR would not only lengthen the ADL, enhancing foraging capacities all else being equal, but it would also decrease maternal maintenance costs and spare energy for fetal growth (Prentice et al., 1989). This would be critical because the placental transfer of both oxygen and nutrients govern intra‐uterine fetal growth (Burton & Fowden, 2015). Given that female Weddell seals have low rates of energy acquisition during their gestational foraging period (Shero, Krotz, et al., 2015), the scope for suppressing metabolic rates may be critical for carrying the pregnancy to term. In contrast, the northern elephant seal (*Mirounga angustirostris*) rapidly accretes tissue during gestation (gaining 70% body mass, as compared with 15% in Weddell seals; Robinson et al., 2012; Shero, Krotz, et al., 2015), and a recent study revealed that reproductive female northern elephant seals made significantly shorter dives during late pregnancy (Hückstädt, Holser, Tift, & Costa, 2018). This suggests that species with greater energy reserves may not require metabolic suppression and energy sparing strategies to protect the developing fetus. Therefore, with climate regime shifts, alterations in prey predictability and abundance may have much more pronounced consequences for Weddell seals, as the energy sparing tactics already in use suggest this species is operating close to its physiological limits. In addition to immediate consequences to pregnancy outcomes (i.e., successful birth vs. fetal loss), variation in nutritional status during prenatal development can have profound life‐long effects on offspring metabolic machinery and overall health and disease (Godfrey, Inskip, & Hanson, 2011).

If long overwinter dive durations are indeed supported by slower O_2_ use rates, this would account for the increased proportion of dives that appeared to exceed the cADL. One disadvantage of using the cADL to assess physiological dive capacity is that it is calculated assuming a fixed DMR, and this highlights the need to consider how both O_2_ stores and use rates are managed by diving mammals. Other physiological changes that may facilitate longer dive durations in the absence of larger TBO_2_ stores include faster processing of anaerobic byproducts (Davis et al., 2004; Thompson & Fedak, 2001). Previous work has shown that these same individuals exhibited increased muscle LDH activity in late winter (Shero, Costa, et al., 2015), coincident with longer post‐dive surface durations. In combination, reproductive females either depressed their DMR over gestation or exceeded their cADL more often (or a combination of both), as compared to seals that were unsuccessful in producing a pup.

Regardless of the proximate cause, that lengthening underwater foraging time is important to pregnant females is suggested by their longer dive durations, the large number of dives exceeding the cADL, and/or the marked drop in DMR suggested by the increase in bADL. As a result, reproductive females spent, on average, an additional 1.09 hr (8.9% more time) each day diving across the winter. Assuming that more time spent diving correlates with higher prey intake, these patterns are similar in magnitude to the 10%–15% increased food intake observed during pregnancy in humans (Rosso, 1987) and likely reflect the additional energetic demands of pregnancy. The increase in dive time across gestation is also similar to the depression in DMR by 9.87%, and the estimated 13.4% increase in energetic demand during pregnancy from these same individual animals (Shero, Krotz, et al., 2015). This all occurs mid‐winter (~June‐August) and coincides with the last trimester of pregnancy, which is the most energetically expensive portion of gestation (20% increase in BMR in humans; Prentice et al., 1989). Indeed, it is during this period of highest gestational energy demand that we see the most marked increases in indices of dive effort at the level of individual dives, dives bouts, and time spent diving. This supports the notion that the altered behavior and increased foraging effort are driven by reproductive costs.

The differences in dive and bout patterns seen in animals of different reproductive class may reflect different foraging niches among individuals with different physiological capacities and energetic demands (Harcourt, Bradshaw, Dickson, & Davis, 2002; Weise & Costa, 2007). All Weddell seals in this study exhibited seasonal shifts in diving behavior, likely due to changes in habitat utilization and overwinter seal movements, prey distribution, and prey‐capture success. Within the Erebus Bay area at the start of tag deployment, local animal dive depths may have been constrained by shallow bathymetry surrounding Ross Island (Eakins & Sharman, 2010; Testa, 1994), or alternatively, animals may have been targeting prey that inhabit shallower layers of the water column. Over winter, Weddell seals travel from Erebus Bay toward regions where more productive Circumpolar Deep Water is advected onto the Ross Ice Shelf up the canyons between the Pennell and Mawson Banks (Burns, Castellini, & Testa, 1999; Goetz, 2015; Testa, 1994). In the spring when the Ross Sea transitions from polar night to high‐light conditions, prey shifts in the water column may force Weddell seals to reach greater depths and exceed the cADL more often to attain prey (Croxall, Everson, Kooyman, Ricketts, & Davis, 1985; McConnell, Chambers, & Fedak, 1992). Animals may also be preferentially diving to attain larger prey items at this time, as the larger size classes of Antarctic silverfish (*Pleuragramma antarcticum*), which comprises the major component of the Weddell seal's diet, reside at >200 m depths (Burns, Trumble, Castellini, & Testa, 1998; Goetz et al., 2017; Hubold, 1984; Hubold & Ekau, 1987). There is evidence that Weddell seals have higher prey‐capture success rates during daylight hours (Fuiman, Davis, & Williams, 2014), which in the study area occur only between September and April, as the sun does not rise from May to August. Therefore, during the winter months, greater energetic gains associated with longer and deeper dives may outweigh the additional costs of diving longer and deeper.

In summary, this study highlights the different foraging and energetic requirements of bringing a fetus to term in a top marine mammal predator. Remarkably, female Weddell seals only exhibit modest mass gains (10%–15% percent) during gestation as compared to other capital‐breeding pinniped species which can almost double their post‐molt mass (Shero, Krotz, et al., 2015). This mass gain remains limited despite significant increases in dive depth and duration, and the total amount of time spent foraging. In combination with estimated aerobic capacity, these changes suggest that seals are either exceeding their cADL fairly often, or reducing their DMR during the winter months. Given that, the total amount of time spent diving each day, and less rest surface periods during foraging bouts, findings suggest that this species may be operating closer to its physiological limits to successfully produce offspring. This would make Weddell seal reproductive output particularly vulnerable to environmental perturbations that would alter prey abundance and predictability.

## AUTHORS’ CONTRIBUTIONS

M.R.S., J.M.B., K.T.G., and D.P.C. conceived the idea for this study and collected the data. M.R.S. analyzed the data and led writing of the manuscript.

## DATA ACCESSIBILITY

All data in this manuscript are accessible through the Antarctic Master Directory. Citation: Costa (2014): Weddell seals as autonomous sensors of the winter oceanography of the Ross Sea. U.S. Antarctic Program (USAP) Data Center. Dataset. https://doi.org/10.15784/600025.
